# The Impact of China's Lockdown Policy on the Incidence of COVID-19: An Interrupted Time Series Analysis

**DOI:** 10.1155/2021/9498029

**Published:** 2021-10-28

**Authors:** Mooketsi Molefi, John T. Tlhakanelo, Thabo Phologolo, Shimeles G. Hamda, Tiny Masupe, Billy Tsima, Vincent Setlhare, Yohana Mashalla, Douglas J. Wiebe

**Affiliations:** ^1^Public Health Medicine Unit, Department of Family Medicine & Public Health, University of Botswana, Gaborone, Botswana; ^2^Family Medicine Unit, Department of Family Medicine & Public Health, University of Botswana, Gaborone, Botswana; ^3^Biomedical Science Department, Faculty of Health Sciences, University of Botswana, Gaborone, Botswana; ^4^Department of Biostatistics, Epidemiology & Informatics, Perelman School of Medicine, University of Pennsylvania, Philadelphia, USA

## Abstract

**Background:**

Policy changes are often necessary to contain the detrimental impact of epidemics such as those brought about by coronavirus disease (COVID-19). In the earlier phases of the emergence of COVID-19, China was the first to impose strict restrictions on movement (lockdown) on January 23rd, 2020. A strategy whose effectiveness in curtailing COVID-19 was yet to be determined. We, therefore, sought to study the impact of the lockdown in reducing the incidence of COVID-19.

**Methods:**

Daily cases of COVID-19 that occurred in China which were registered between January 12th and March 30th, 2020, were extracted from the Johns Hopkins CSSE team COVID-19 ArcGIS® dashboards. Daily cases reported were used as data points in the series. Two interrupted series models were run: one with an interruption point of 23 January 2020 (model 1) and the other with a 14-day deferred interruption point of 6th February (model 2). For both models, the magnitude of change (before and after) and linear trend analyses were measured, and *β*-coefficients reported with 95% confidence interval (CI) for the precision.

**Results:**

Seventy-eight data points were used in the analysis. There was an 11% versus a 163% increase in daily cases in models 1 and 2, respectively, in the preintervention periods (*p* ≤ 0.001). Comparing the period immediately following the intervention points to the counterfactual, there was a daily increase of 2,746% (*p* < 0.001) versus a decline of 207% (*p* = 0.802) in model 2. However, in both scenarios, there was a statistically significant drop in the daily cases predicted for this data and beyond when comparing the preintervention periods and postintervention periods (*p* < 0.001).

**Conclusion:**

There was a significant decrease the COVID-19 daily cases reported in China following the institution of a lockdown, and therefore, lockdown may be used to curtail the burden of COVID-19.

## 1. Introduction

The recognition of a possible outbreak followed the identification of a cluster of cases presenting with a rare type of pneumonia [1]. These cases had both epidemiological and geographical ties to the Huanan seafood market in Wuhan, Hubei province, China [2, 3]. Samples from these patients later revealed a novel type of coronavirus known as SARS COV-2 [4]. A virus closely associated with those that cause severe acute respiratory syndrome (SARS) and Middle East Respiratory Syndrome (MERS) [5, 6].

The few weeks that followed saw more cases being detected in the Hubei province, with Wuhan city having the highest number of cases [7]. The cumulative incidence increased exponentially daily reaching a few hundred in less than three weeks [8]. The situation needed containment especially with evidence of local transmission taking place in neighbouring provinces [9].

On January 23rd, 2020, the government of China imposed a lockdown on Hubei province in an effort to control the spread of the disease [10]. The lockdown resulted in restrictions on movement among residents of the province requiring all to stay indoors during this period. There were mixed reactions to this intervention with some labelling it as extreme especially as social support was not guaranteed [11, 12]. While China has recently reported a decline in the incidence of COVID-19, it is not clear what impact the lockdown has had on this decline.

This study sought to quantify the impact that China's lockdown policy had in reducing the incidence of COVID-19 using interrupted time series methods.

## 2. Methods

Cases of COVID-19 in China as reported daily were extracted from the Johns Hopkins CSSE team ArcGIS dashboard [13] for periods between January 12th and March 30th, 2020. These figures were then matched to China's projected population for 2020, estimated at 1,408 526 449 according to the United Nations' population division. The daily occurrences of cases were then calculated and used as data points in the time series analysis using 23rd January as the intervention point (Tables 1 and 2, model 1). Additionally, because we did not expect the impact of the lockdown to start immediately, we modelled the data where we carried forward the date intervention to 6th February (Tables 3 and 4, model 2); the length of time corresponding to the maximum incubation period of 14 days for someone who was exposed on or about 23rd January 2020.

Data were modelled as a single-group interrupted time series analysis without a comparator for both scenarios using the *itsa* syntax in Stata®. The level of change and the trajectory of change following the intervention obtained via ordinary least-squares [14] regression estimates were evaluated yielding *β*-coefficients and Newey-West standard errors. The Cumby-Huizinga test for serial autocorrelation using *actest*, *lags* in Stata [15, 16] was employed as a postestimation command following the OLS regression.

## 3. Results

**Figure 1:**
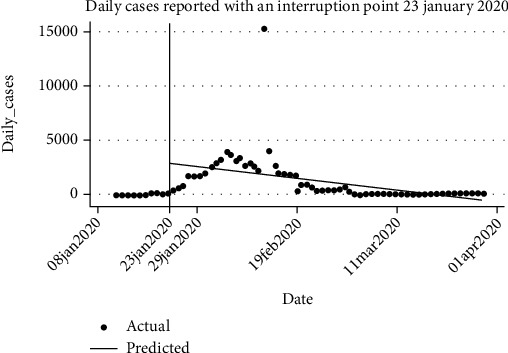
Daily cases reported over time showing a 23rd January interruption point (model 1).

**Figure 2:**
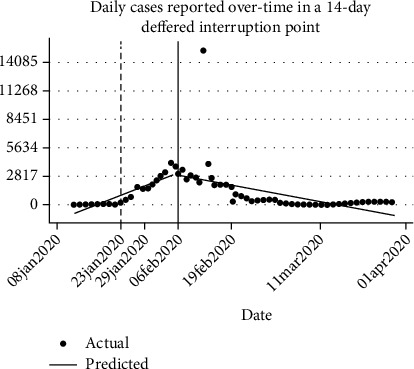
Daily cases reported over time showing a 14-day deferred interruption point from 23 January to 6th February 2020 (model 2).

Seventy-eight data points were used in the series with 11 (14.10%) and 25 (32.05%) data points used in the preintervention period in Figures 1 and 2, respectively. In the preintervention phase, Table 1 shows a 11% (95%CI = 3.95, 18.23) increase in the occurrence of daily cases while Table 3 shows an astronomical increase of 163% (95%CI = 110.29, 216.06). When comparing the period immediately following the intervention versus the counterfactuals for both scenarios, the data showed an overwhelming 2,746% (95%CI = 1142.18, 4350.70) increase in daily cases in Table 1 against the counterfactual while Table 3 data shows a 207% decline in the occurrence of daily cases; however, the latter was not statistically significant (*p* = 0.832). When measuring the magnitude of change between the pre- and postintervention periods, we obtained a 60% (95%CI = 25.98, 95.51) decline and 240% (95%CI = 167.97, 313.01) decline in the occurrence of daily cases for Tables 1 and 3, respectively.

## 4. Discussion

Prior to this study, there was a question about the effectiveness of lockdown as a strategy for the containment of the spread of COVID-19 [12]. The findings herein reveal an increasing occurrence of daily cases of COVID-19 in the preintervention period, as at the time there were no restrictions on movement of people and goods across China before the lockdown and so disease transmission continued to flare. This significant increment of new cases daily would potentially have reached epic proportions and had the lockdown intervention not been instituted. This evidence is supported by the rising numbers of daily cases reported when comparing the postintervention cases and the counterfactual scenarios.

There was a statistically significant predicted rise in the number of daily cases in model 1 (Table 1) in the period immediately following the intervention. This was to be expected as at the time there may have been many other people who may have been infected and were yet to be diagnosed, but more importantly, the effects of such an intervention would take time to show, hence our modelling of a deferred intervention point. When comparing the preintervention and postintervention periods, while a nondeferred intervention point (Table 1) showed a modest decrease of 60% in the occurrence of daily cases, Table 3 modelled on deferred intervention point shows an overwhelming 4 times the decline in daily cases. This reduction thus indicates that the lockdown policy not only had a positive impact in reducing the incidence of COVID-19 but also resulted in an accelerated way of reversing the situation. The finding that China's COVID-19 incidence is decreasing is consistent with findings from other studies [17, 18].

A more specific yet less stringent intervention is the notion of social distancing which has been implemented its effects evaluated in a several settings, alongside other interventions. Several studies evaluating the effectiveness of the social distancing policy using the interrupted time series analysis methods have been published [19]. Saki et al. studied the effects of social distancing policy on the incidence and deaths of COVID-19 in Iran. The findings were consistent with ours, where they found a decrease in the number of new cases occurring and a decreasing trend after the implementation of the social distancing policy. Additionally, Alimohamadi et al. also demonstrated the positive effects of the social distancing policy in curbing the incidence and mortality from COVID-19 in Iran. Both studies corroborate the findings from China in the early days of the pandemic. Of note is that the impact estimated herein is only conservative as there was a change in the case-definition of a COVID-19 case in mid-February in China from symptoms and a positive test to just symptoms [20]. An action that could result in the artefactual increment of COVID-19 cases is reported.

Since we were evaluating the impact of a large-scale intervention using data before and after an intervention, interrupted time series methods were suitable for this purpose. These methods are powerful methods of high validity since they control for common threats of confounding which exist in other observational studies [21] therefore are a good compromise when it is not feasible or unethical to randomize units/subjects [22]. With a distinct time of intervention when the lockdown was announced and factoring in an estimated period of diffusion, we believe that approach enabled us to mimic the truth about the evolution of the disease in China. Furthermore, sensitivity analysis revealed the validity of our model up to 4 lags for both scenarios.

As more countries have started having cases of COVID-19, they will be looking for effective strategies to control the outbreak in their settings. This paper demonstrates the effectiveness of a lockdown strategy and that it may be necessary to prevent more cases from occurring during an outbreak.

There are limitations that need noting in this study. First, we cannot rule-out the possibility of other interventions that could have contributed to the change; though so far, none have been published or reported. Secondly, we relied on secondary data as it was being reported, and we only had limited data points to model with before the intervention as it was only the beginning of the outbreak. A review and analysis of more recent data in other settings to evaluate the impact of lockdown strategy in reducing the burden of COVID-19 cases are needed.

## 5. Conclusion

There is evidence that the lockdown policy introduced by China in containment of COVID-19 has completely reversed the occurrence of COVID-19 cases reported daily. Lockdown policy presents a viable option of burden of COVID-19.

## Figures and Tables

**Table 1:** Interrupted time series ordinary least-squares regression output at 23rd January 2020 intervention point (model 1).

	*β*-Coefficient	Std error	*p* value	95% CI
Preintervention	11.09	3.58	0.003^∗^	3.95, 18.23
Immediately postintervention vs. counterfactual	2746.44	805.13	≤0.001^∗^	1142.18, 4350.70
Pre- vs. postintervention	-60.75	17.45	≤0.001^∗^	-95.51, -25.98

**Table 2:** Postlinear trend regression output from 23rd January 2020 interruption point (model 1).

	*β*-Coefficient	Std error	*p* value	95% CI
Postintervention linear trend	-49.66	15.89	0.003^∗^	-81.33, -17.99

**Table 3:** Interrupted time series ordinary least-squares regression output at 14-day deferred intervention (model 2).

	*β*-Coefficient	Std error	*p* value	95% CI
Preintervention	163.18	26.54	≤0.001^∗^	110.29, 216.06
Immediately postintervention vs. counterfactual	-206.95	971.17	0.832	-2142.06, 1728.16
Pre- vs. postintervention	-240.49	36.40	≤0.001^∗^	-313.02, -167.97

**Table 4:** Postlinear trend regression output using 6th February 2020 interruption point (model 2).

	*β*-Coefficient	Std error	*p* value	95% CI
Postintervention linear trend	-77.32	24.60	0.002^∗^	-126.34, -28.30

## Data Availability

The data are freely available online at https://www.arcgis.com/apps/dashboards/bda7594740fd40299423467b48e9ecf6 and the United Nations' population division website.

## Additional Points

*What Is Already Known on the Subject*. Evidence for the effectiveness of “lockdown” as a strategy in controlling the surge of COVID-19 was lacking, despite many countries continuing to adopt it. *What the Study Adds*. This study illustrates the effectiveness of the “lockdown” strategy or policy in controlling the surge in cases of COVID-19 through the use of quasi-experimental methods on early data generated from China. *Policy Implications*. Lockdown policy or strategy could be adopted as an effective means of reducing the COVID-19 incidence. *Patient and Public Involvement*. Since this is an ecological study looking at aggregate data, it was not possible to involve patients or the public in the design, conduct, and reporting of our research.
